# Contiguous US summer maximum temperature and heat stress trends in CRU and NOAA Climate Division data plus comparisons to reanalyses

**DOI:** 10.1038/s41598-018-29286-w

**Published:** 2018-07-24

**Authors:** Richard Grotjahn, Jonathan Huynh

**Affiliations:** Atmospheric Science Program, University of California, Davis, CA 95616 USA

## Abstract

Warming is a major climate change concern, but the impact of high maximum temperatures depends upon the air’s moisture content. Trends in maximum summertime temperature, moisture, and heat index are tracked over three time periods: 1900–2011, 1950–2011, and 1979–2011; these trends differ notably from annual temperature trends. Trends are emphasized from two CRU datasets (CRUTS3.25 and CRUTS4.01) and two reanalyses (ERA-20C and 20CRv2). Maximum temperature trends tend towards warming that is stronger over the Great Lakes, the interior western and the northeastern contiguous United States. A warming hole in the Midwest generally decreases in size and magnitude when heat stress trends are calculated because the region has increasing moisture. CRU and nearly all reanalyses find cooling in the northern high plains that is not found in NOAA Climate Division trends. These NOAA trends are captured better by CRUTS401. Moistening in the northeast amplifies the heat stress there. Elsewhere the moisture trends are less clear. Drying over northern Texas (after 1996) in CRUTS401 translates into decreasing heat stress there (less so in CRUTS325). Though other reanalyses are not intended for long-term trends, MERRA-2 and ERA-Interim match observed trends better than other reanalyses.

## Introduction

Concern is growing about warming near-surface temperatures^1–4^. Impact studies sometimes emphasize maximum temperatures^5^ but it is well known^6^ that other factors such as moisture, sun exposure, and lack of wind amplify human and animal discomfort and mortality. Many metrics^7^ have been developed to measure heat stress. This study calculates trends in daily maximum temperature (Tmax) during June-August (JJA) then adds humidity in two metrics. A metric common in agricultural contexts (dairy^8^) is the temperature-humidity index (THI). A human discomfort metric routinely posted on public weather information sites is the apparent temperature or heat index^9^ (HI). This study examines trends in these metrics during summer.

Long term, quality-controlled datasets^10–12^ are used to calculate trends. These datasets generally do not include moisture needed for THI and HI. CRU325^13^ and CRU401^14^ data contain high resolution monthly mean Tmax and near-surface water vapor data making them the focus of this study. Time and space intervals may be larger than needed for some applications. Other variables (wind speed and radiation) at the time of maximum temperature may be needed. If so, one might consider a reanalysis, providing motivation to show corresponding reanalysis trends.

Many instruments observe the atmosphere with widely varying accuracy and irregular distribution in time and space^15^. Consistency when objectively analyzing observations onto a regular grid should facilitate calculating trends^16^.

CRU325 and CRU401 are two versions of monthly station data interpolated^17^ onto a 0.5° latitude/longitude grid. Tmax is a ‘primary’ variable in these data while the moisture variable available, vapour pressure, is a ‘secondary’ variable since it includes ‘synthetic’ vapor pressure estimated from minimum temperature^18^ on a coarse grid in regions with too few station measurements of vapor pressure. CRU325 uses triangulated linear interpolation^17^ whereas CRU401 uses angular distance weighting^19^.

NCD data^20^ provide a baseline for observed changes^21^; here NCD provides a check on the CRU datasets. GHCN^22^ station data are interpolated to 344 NOAA Climate Division shapefile areas. GHCN data have conflicting trends in adjacent stations, as illustrated in Supplementary Fig. S1. NCD data make extensive homogeneity adjustments for: missing dates, local geographic effects^23^, instrument changes, and changes in observing time before forming monthly values assigned each area. NCD does not include atmospheric moisture content precluding calculation of HI or THI.

Reanalysis 20CRv2^16^ only uses surface pressure observations even when additional observing platforms become available, but the number of observing stations increases by >10 times from 1901 to 2011. ERA-20C^24^ is also designed for long-term trends. In contrast, a reanalysis like NNRA1^25^ uses a consistent model but incorporates new data sources (e.g. satellites) as they become available, resulting in artificial jumps^25^ in the time series of variables thereby casting doubt on a trend analysis^26^.

These five datasets: CRU325, CRU401, NCD, 20CRv2, and ERA-20C are emphasized in this report. Other reanalyses: ERA-I^27^, MERRA2^28^, CFSR^29^, NARR^30^, NNRA1^25^, and NDRA2^31^ are shown only for comparison as motivated above.

## Thermal and Moisture Trends Context

Changes in station location, instrumentation, reporting variables, and reporting time create inhomogeneities^20^ that reduce the accuracy of trend calculations^32,33^. From 1984 (reaching 60% of stations by 1990) liquid glass max/min thermometers were replaced with thermistors (MMTS) and summer maximum temperatures dropped ~0.4 C over the CONUS^34^. MMTS maximum temperature may be more accurate^34^; however, the change is large enough to add cooling to Tmax trends during our shortest period. Station exposure needs adjustment^35^ as some sites show a false Tmax trend of ~0.5 C from 1980–2008. Minimum temperatures increased ~0.3 C on average over the CONUS with MMTS changeover^34^ but reporting time change (from afternoon to morning) had the opposite effect^35^. Adjusting for the time of day reporting creates warmer mean temperatures^20^ with notable exceptions: much of Nevada and adjacent climate divisions in Utah, Idaho, and Arizona, most of North Dakota, Iowa, Missouri, and Oklahoma. Spurious elevated minimum temperatures may impart spurious moistening of ‘synthetic’ vapor pressure trends where used in CRU data if not adjusted. During 1981–2010^20^ a quarter of the observing sites are missing data on half the days! Simple averages over regions when data are missing introduce biases^20^. Differences between station data versions^20^ or between carefully-constructed datasets^3^ are much smaller than differences shown here between reanalyses.

Trends in annual average temperature^21,36,37^ find a cooling region termed a ‘warming hole’. This hole’s location varies in annual average data, being generally in the southeastern^21,33,36^ CONUS though others place it more centrally^38,39^. Disagreement arises from different time periods for the trend^2,3,10,33,39–41^. Higher resolution^10^ expands cooling in the southeast and enhances warming in the interior west. High temperatures in the 1930s cause 1930–1990^42^ and 1930–1950^43^ trends to have cooling over most of the US. Such results motivate consideration of different time periods.

This warming ‘hole’ expands^2,10,39,43,44^ into the Midwest during JJA. However, for 1979–2005 only warming in the far west passes a 5% significance test^2^. GISSTEMP show a similar pattern but have little or no cooling during 1950–2009^10^. JJA temperatures in HadCRUTv show cooling^39^ over more of the Midwest extending to the Canadian border as well as over California (1976–2000). Midwest cooling has been ascribed to low frequency variation of equatorial central Pacific sea surface temperatures and convection^38,45–47^. The summer warming hole may arise from increased moisture convergence by Midwest low level jet changes that enhanced precipitation in turn enhancing shading by clouds and elevating soil moisture and thus evapotranspiration^48^. Aerosols cooling the North Atlantic^42^ may enhance this latter effect. A positive trend in precipitation is correlated with a negative trend of early summer hottest temperatures^49^.

Tmax has different trends from daily mean temperature. Station data have cooling over most of the eastern half of the CONUS with warming elsewhere^49^ in May-June Tmax (1950–2006). Warming daily minimum temperatures partially compensates cooling of daily Tmax across most of the Midwest^50^ when averaging differences between successive decades during 1960–1999. CRU Tmax trends^51^ are cooling over the Central and Northern Plains^52^ during JJA (1979–2000) but not annual averages. Another study^53^ finds warming of JJA Tmax over the CONUS except for Florida and the Montana-North Dakota border; with stronger warming over the upper Great Lakes and areas from Texas to Nevada. CRU data have near zero trend^51^ over the southeast, cooling for a Texas grid cell, and warming elsewhere (1950–2004). During 1950–1995^54^, nearly all of the CONUS has cooling except the far west.

Moisture has fewer observations^15^ than temperature. Annual mean data^55^ have significant increases in specific humidity (q, and relative humidity, RH) over the eastern and northern 3/4 of the CONUS with some reduction over the southwest deserts (1976–2004). However, HadISDH^56^ data (1975–2010) have q increasing less in the eastern CONUS^57^ resulting in RH decreases over most of the CONUS, especially over southwest deserts. Other trends: 1973–1999^58^ and 1973–2003^59^ find q increasing over the eastern US but decreasing over the northwest. Comparing 5-year averages (1999–2003) of HadCRUH and ERA-Interim data^60^ find generally similar patterns of q anomalies from 1989–1998 means. Summer moisture trends (1973–1999^59^; 1973–2003^61^) show more widespread moistening over the CONUS and smaller regions of drying out west relative to annual trends. Summer trends (1979–2013)^53^ increase RH in Washington, parts of the northern Plains, northeast, and Florida with drying elsewhere especially from Nevada across to Louisiana.

Trends of days/decade of extreme HI values at 187 GNCN stations (1949–2010)^62^ significantly increase (1 to 3 or more) over Florida, parts of Texas and the Great Basin and the northern Rockies. During 1948–2012, the number of days, events, and duration of high HI events increase with the bigger changes in Gulf of Mexico states and interior western CONUS^63^.

## Trends in CRU, NCD, 20CRv2, and ERA-20C

### Maximum Temperature Trends

**Figure 1:**
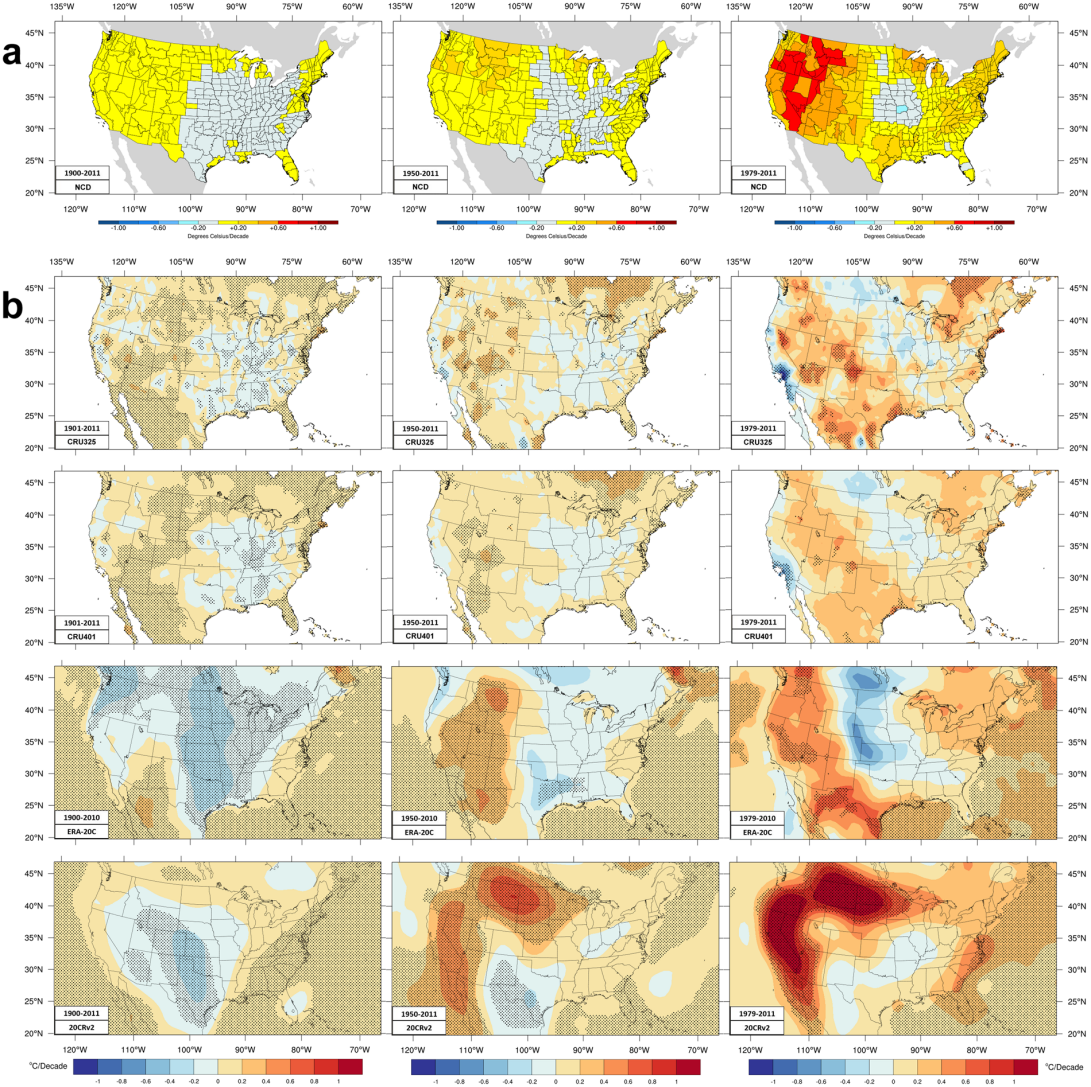
Trends in summer maximum temperature. Color shading denotes the indicated ranges of June-August (JJA) mean maximum temperature trends during three time periods. The longer time periods are on the left column, the intermediate time periods are the center column, the shorter time periods are the right column. Shading denotes data passing a Mann-Kendal significance test at the 5% level. (**a**) Trends in NCD monthly mean data. (**b**) Trends in CRU (CRU325 and CRU401) and the two long-term reanalyses (ERA-20C and 20CRv2) daily data. The units are K/decade.

Figure 1 shows Tmax trends over three time periods: 1901–2011, 1950–2011, and 1979–2011 that are referred to as the longer, intermediate, and shorter time periods, respectively.

Over the longer period, NCD shows general warming in the west with no values greater than 0.2 K/decade. These small trends have similar magnitude as annual temperature trends^20^ and a consequence of extraordinarily warm summers in the central and western areas during the 1930’s. Most of Oregon, Montana, Wyoming, Utah, and Arizona are between 0.1–0.2 K/decade. There is slight warming (<0.1 K/decade) in the northeastern and mid-Atlantic states with some coastal zones warming by 0.1–0.2 K/decade. The general pattern of central and southeastern CONUS cooling with warming elsewhere (including Florida) is common among NCD, CRU325, and CRU401. CRU325 has areas of cooling and too little warming west of 105 W compared with warming there in NCD. Many areas west of 105 W that have CRU325 cooling are areas where NCD warming is <0.1 K/decade. In general, the CRU401 pattern is smoother and the peak values less than in CRU325. CRU401 also have smaller but more spatially-connected regions passing the significance test; these regions tend towards good agreement with NCD data, though central California is an exception. Warming over Rocky Mountain States has less spatial variation in CRU401 and matches better NCD trends. The cooling in central and southeastern CONUS is more uniform in CRU401 and matches better NCD data, notably matching the higher rates of cooling (−0.1 to −0.2 K/decade) in Nebraska and lower Ohio River areas. Both CRU patterns present higher warming rates (0.1–0.2 K/decade) along the mid-Atlantic and northeast much like NCD trends. CRU data tend towards slightly too much warming over Florida, Kansas, Oklahoma, and southern Texas.

Over the intermediate time period, CRU401 data remain smoother than CRU325 and match better the sign of NCD trends. NCD trends tend to be larger in the northern Rockies than CRU values; though CRU325 has higher peak values, they do not align consistently with higher NCD values. Areas passing the significance test are noticeably smaller in CRU401 though again, these are areas matching NCD. NCD warming trends are largest in the northern Rockies, Oregon, and around Lake Superior, ranging from 0.2–0.3 K/decade. CRU trends have significant warming in parts of those regions. The CRU trends along the west coast again differ from NCD values; particularly true for coastal Southern California and Oregon. Many coastal stations may define the trend for a larger region in CRU data whereas corresponding climate division NCD averages downplay those stations in favor of inland stations because the marine influence does not usually extend far inland^20^. The warming hole is similar to before, but smaller in size and the strongest cooling trends are generally reduced. The cooling region also shrinks in CRU trends, mainly in the southeast (some regions being significant). NCD finds cooling over all of Missouri which is better captured by CRU401. CRU trends continue warming over Oklahoma, Kansas, and southwest Texas where NCD finds cooling.

Over 1979–2011 trends become larger and differences become clearer between CRU325 and CRU401. Disagreements with NCD trends are also more pronounced. NCD warming trends become quite large west of 105 W. Much of western Montana, the Great Basin, Oregon, and Southern California deserts have very high warming rates (0.6–0.8 K/decade); southern Oregon and northwest Nevada and the upper Snake River in Idaho trends range from 0.7–0.8 K/decade. CRU data also have higher warming trends out west, though the locations of peak warming sometimes match sometimes miss greater warming in NCD. CRU401 trends are again smoother and peak values less than in CRU325. The areas passing the significance test again shrink as the period shortens. Warming trends are high (0.4–0.5 K/decade) around the upper Great lakes in NCD, a property better reproduced in CRU401. CRU data have smaller warming (0.1–0.3 K/decade) in the northeast (and southern Appalachian Mountains) than does NCD (0.2–0.5 K/decade). Contrary to a cooling trend during the other time periods, Texas, especially along the coast, has clear warming (0.1–0.4 K/decade) in NCD and both CRU datasets. The warming hole has continued to shrink in area, though peak cooling trends (in Missouri) are larger (up to −0.2 to −0.3 K/decade). The Midwest warming hole shrinks in CRU, though the area of greater cooling tends to be further north (in CRU401 and CRU325) and further west (in CRU325) than in NCD. Bucking previous trends, a band across Florida has slight cooling (−0.1–0 K/decade) in NCD that is less clear in CRU. Similarly, Washington’s Olympic peninsula has cooling in NCD and CRU325 trends. The disagreements between NCD and CRU along the California coast are more pronounced, and those are the only CRU cooling trends passing the significance test. Disagreement arises along the central Canadian border where CRU have a cooling (−0.1 to −0.2 K/decade) peak values where NCD has warming (0.1–0.3 K/decade).

These results are similar to those in previously published work. Examining^64^ three other interpolated station datasets for 1979–2010 finds Tmax warming (~1/4 to ~1/2 K/decade) over the western CONUS while the northern Midwest has some cooling (~1/4 to ~1/2 K/decade). Like CRU, the cooling extends across the Canadian border. Analyzing trends (1980–2015)^65^ at GHCN^22^ stations finds warming of the median Tmax by up to + 1 C in the northwestern US and cooling by more than −0.5 K in the central US (near 90 W/40 N) with lesser cooling extending to the Atlantic and southeast.

**Figure 2:**
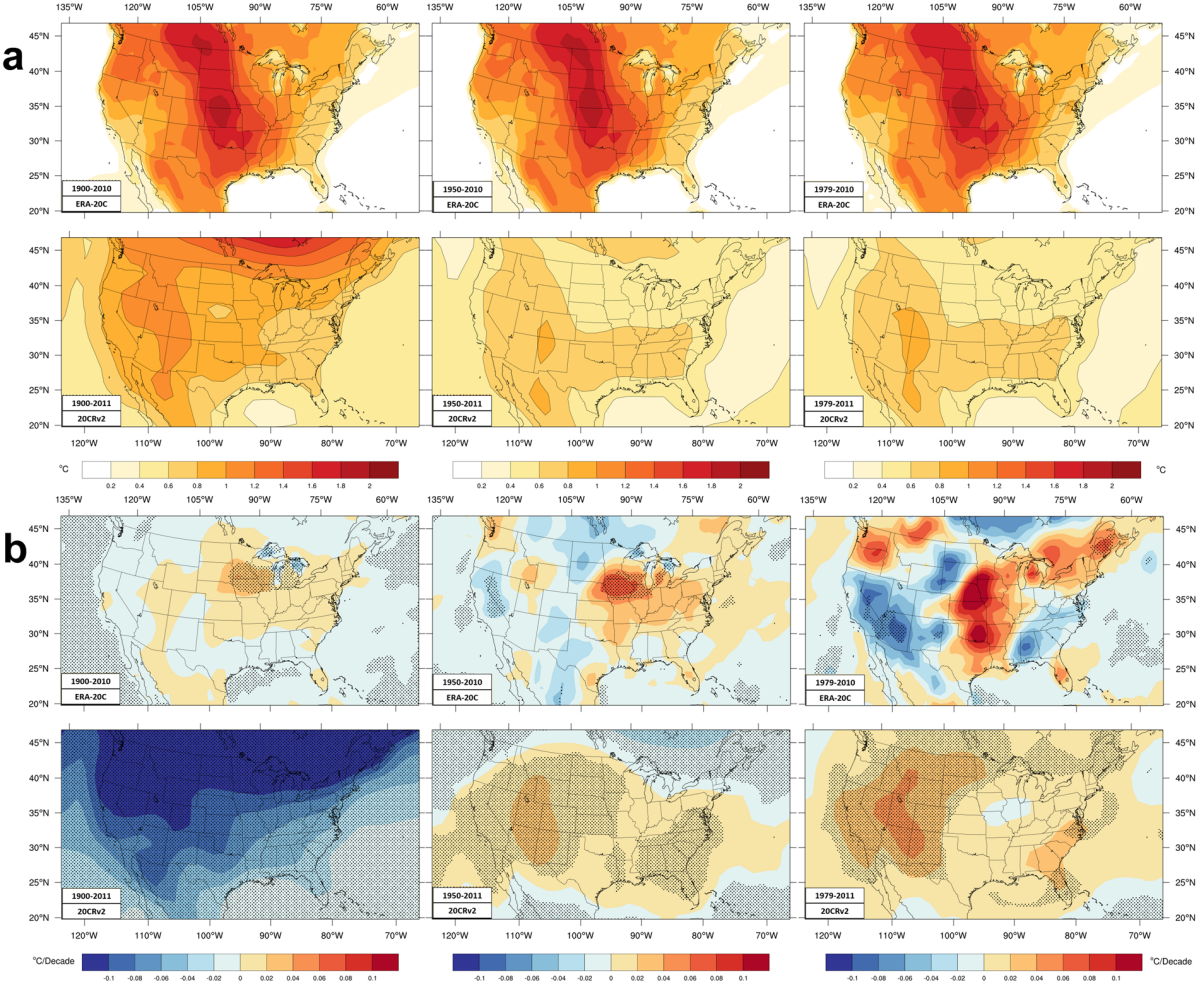
ERA-20C and 20CRv2 ensemble spread during the three time periods. (**a**) The ensemble spread (measured by the standard deviation) of the daily mean temperature values. Units are C. (**b**) The ensemble spread trend. Blue colors denote decreasing spread over the period, while warm colors indicate increasing spread among the ensemble members.

Figure 1 includes ERA-20C and 20CRv2 trends. ERA-20C are from an ensemble of 10 model integrations^66^. 20CRv2 are from a 56-member ensemble. Ensembles allow analysis of average differences between ensemble members. Figure 2 shows the ensemble spread averaged over the periods and its trend during the time periods.

For the longer period, both reanalyses have cooling over the middle CONUS. ERA-20C extends cooling too far west, including stronger, significant cooling in the northwest that is generally opposite to other maps shown, though the spread is somewhat larger there. ERA-20C has largest spread in the central and northern plains and the spread increases there over time. 20CRv2 cooling extends too far west and not far enough east, having significant cooling over the central Rockies, where CRU (significant) and NCD trends have warming. The Rockies and Great Basin have the largest spread in 20CRv2 while the trend indicates declining variation between ensemble members over the entire CONUS. The 20CRv2 spread is clearly greatest earlier in the period. The larger cooling rates (−0.2 to −0.4 K/decade) in both reanalyses’ trends are larger than found in the NCD and CRU data. Neither reanalysis has trends matching the interpolated station datasets well. Shading indicating regions of significant trends match some places (e.g. Texas) and do not match trends other places (e.g. Colorado).

During the intermediate period the two reanalyses have trends generally matching better CRU and NCD trends than they do for the longer period. Both ERA-20C and 20CRv2 match NCD having greater warming in the northern Rockies, perhaps better than the CRU data, though these reanalyses trends are much larger than the NCD trends. Warming around the Great Lakes and southeast developed in 20 CRv2 but not so much in ERA-20C. However, ERA-20C captures the area of the warming hole better than 20 CRv2 even though the ensemble spread and the spread trend are greater there for ERA-20C. ERA-20C trends have significant warming over the interior and west and significant cooling in the south-central CONUS. Cooling in ERA-20C elsewhere in the central US and warming over Mid-Atlantic States do not pass the significance test but have similar sign as NCD and CRU values. 20CRv2 trends have a prominent ‘Γ-shaped’ region of very strong warming near the west coast and across the northern Plains. The portion of the ‘Γ-shape’ north of 42 N arguably agrees better with NCD trends than the other datasets, though the warming rate exceeds NCD. While 20 CRv2 trends somewhat match in sign over those regions, significant cooling over New Mexico clashes with the other results, notably, the ensemble spread and spread trend are larger there. Elsewhere, 20 CRv2 trends have weak cooling in the Ohio River Valley, consistent with the other datasets.

During the shorter time period both reanalyses have stronger warming over western mountains, Great Basin, and west coast. Neither has the cooling along the west coast found in CRU trends but not in NCD trends. ERA-20C matches the amplitude of NCD and CRU trends data better in the west. Both reanalyses have stronger warming in the Great Lakes and northeastern US. The warming hole in the central US is better handled in area and amplitude by 20CRv2. ERA-20C has too strong cooling and places the peak cooling too far west, though these values are not significant and the ensemble spread is largest there. The spread is increasing in the central CONUS for ERA-20C. 20CRv2 sustains cooling over New Mexico that is not present in the other datasets, where the ensemble spread increases over time and the trend is not significant. The 20CRv2 ‘Γ-shaped’ region has even stronger trends, again passing the significance test, and again stronger than NCD trends. This excessive warming, in a precursor to the 20CRv2 data, has been seen in annual mean temperature trends^67^ (1979–2008). 20CRv2 has very strong warming over Montana and the Dakotas where CRU and ERA-20C (but not NCD) have consistent cooling.

### Moisture Trends

**Figure 3:**
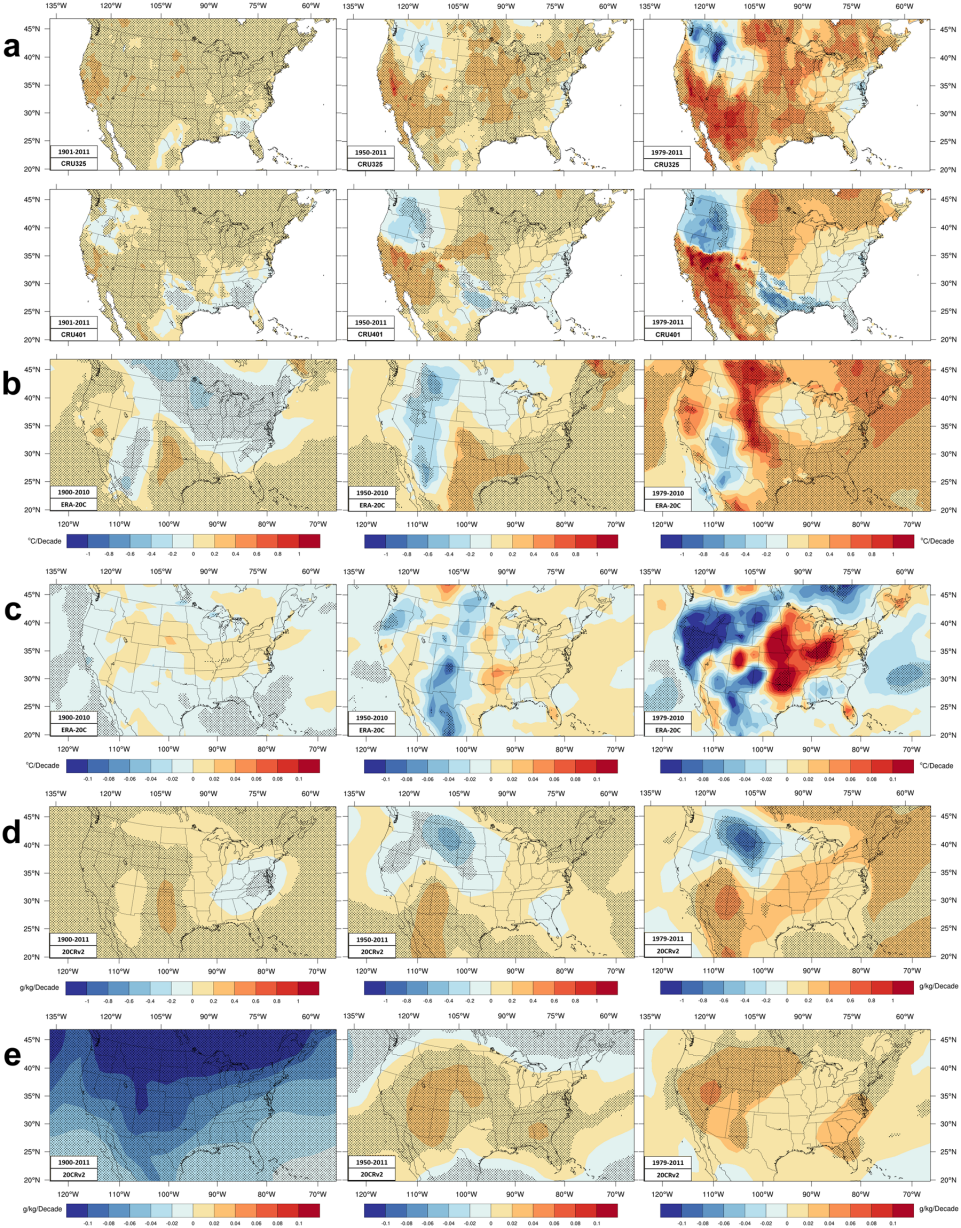
Trends of daily mean moisture variables. Similar format as Fig. 1 with three columns, each for a different time period. (**a**) Trends in seasonal (JJA) averages of daily mean dew point (Td). (**b**) Trends in seasonal averages of daily maximum Td in ERA-20C data. (**c**) Trends in the standard deviation of ERA-20C ensemble seasonal means of Td. The color scales for (**a**–**c**) are K/decade. (**d**) Specific humidity trends in 20CRv2 and (**e**) corresponding trend in standard deviation among ensemble members where the color scale shows the change per decade of gm water vapor per kg of air.

Figure 3 presents moisture variables trends. Moisture in CRU and ERA-20C is expressed using dewpoint (Td). 20CRv2 data includes q. Trends in the standard deviation of Td and q are shown (Fig. 3c,e) for the reanalyses.

During the longer period, significant moistening covers most areas in CRU. Larger trends of daily mean Td occur over mountainous and central California. CRU325 has larger moistening over the high plains. Both CRU have significant decreasing Td in part of the southeast, with a larger area found in CRU401 data. CRU401 has decreasing moisture over much of the northwest, and northern Texas that becomes more prominent in later periods.

During the intermediate period, similarities and differences in longer-period trends become more pronounced. CRU325 has significant moistening of the southwestern CONUS and drying (not significant) over Washington and Idaho. CRU401 has similar pattern south of ~41 N except for a sharp boundary to a larger area of drying over the northwest. CRU325 has moistening over the eastern two-thirds of the country except small portions near the Atlantic coast. CRU325 trends are stronger over the southwest, extending into Oregon, the Midwest and upper Great Lakes, and parts of the northeast. CRU401 also differs by (non-significant) drying over much of the southeast and an odd significant drying over northern Texas.

During the shorter period, trends are further amplified. The southwest experiences the largest moistening in CRU. Both CRU have significant moistening over the Midwest and northern plains and part of New England. CRU325 again has significant moistening over much of the southern and Gulf Coast states. Drying over parts of the northwest is a feature of more recent years in the datasets as the respective intermediate period trends are now stronger. Drying over Texas and Louisiana is also a recent trend.

These two larger areas of drying in CRU401 prompted further investigation. The drying trend from northern Texas across to the Gulf coast may be an artifact in CRU401 though a similar feature appears in PRISM trends^68^. Prior to 1997 contours of JJA vapor pressure smoothly curve across the south central US roughly parallel to the Gulf coast in a similar pattern each year in both CRU. However, from 1997 onwards each of those contour lines in CRU401 ‘cut-in’ over northern Texas to the Mississippi delta while other segments of those curving contours have only small variations; this cut-in appears in all later years but not in earlier years (Supplemental Fig. S2). The ‘cut-in’ contours overlie the drying trend location for CRU401 (Fig. 3a). The cause of the western CONUS sharp change is not so visible since very low vapor pressures prevail over the Great Basin though CRU401 is dryer than CRU325 data after 1999.

During the longer period the two reanalyses agree on significant stronger moistening over the southern high plains, moistening over the far west and Gulf coast, and drying over Mid-Atlantic States. However, ERA-20C and 20CRv2 find opposite trends over the southern Rockies and from the northern Plains across to the northeast CONUS. Though only ERA-20C trends of daily maximum Td (Fig. 3b) are significant where the reanalyses disagree, the 20CRv2 trends of q (Fig. 3d) in those areas match better the CRU trends. The ensemble spread (Fig. 3e) is large early on in the 20CRv2 data and declines over time; the spread trend is also larger over the west where the two reanalyses disagree.

Over the intermediate period, both reanalyses moisten the south-central US (passing significance tests over much of Texas). However, peak moistening is further west in 20CRv2 and aligns better with CRU trends. In contrast, ERA-20C has drying over southwestern deserts unlike the other three datasets. Elsewhere, ERA-20C and 20CRv2 find drying in the northern Rockies and northern Plains; the former matches CRU data but not the latter. ERA-20C has significant moistening in the southeast but 20CRv2 has a mixture of trends in this area where the CRU datasets also disagree. All four datasets moisten the northeast.

Moisture trends during the shorter period differ markedly between the reanalyses, though both moisten east of 85 W with significant larger moistening over the northeast. They both moisten the southern plains and Gulf coast, though only ERA-20C trends are significant. West of ~105 W the two reanalyses generally have opposite trends, ERA-20C dries some southwest deserts where 20CRv2 has maximum moistening. Similarly, ERA-20C has peak moistening in the northern high plains where 20CRv2 has peak drying. However, the ensemble spread in 20CRv2 increases everywhere, especially over the Great Basin. For ERA-20C, the ensemble spread diminishes in the west but strongly increases over the Midwest

There is more disagreement between these moisture trends than was found for Tmax. One reason may be the greater sensitivity of *Td* and *q* to changes in instruments^69^ that occurred around 1960, 1985, 1995, and 2005 causing ‘breakpoints’ in station continuity at 10% or more of stations. Dewpoint during 1947–2010 generally increases at individual stations^69^ east of ~105 W, significantly increasing at several stations between 37–46 N; magnitudes vary but many of these stations have ~0.4 K/decade increases. To the west, stations in the northern high plains and Great Basin have inconsistent Td trends; magnitudes vary but range from −0.8 to + 0.8 at different stations. These station trends do not match either intermediate CRU pattern fully, but CRU325 and 20CRv2 come closer in signs and magnitudes. The primary disagreements between CRU325 and the station trends^69^ are where stations have drying south of ~41 N and where stations have moistening in the northwest. Gridded HadCRUH q trends are somewhat different over 1973–2003 having decreases^59^ along 40–45 N from near the west coast to the central CONUS with more drying near the west coast; moistening near North Dakota and perhaps elsewhere to the east. Comparing q over 1973–1999 relative to the 1974–1999 average^61^ shows decreases inside small areas near 40–45 N in the western and central US and moistening elsewhere in HadCRUH^59^. These HadCRUH trends look more similar to 20CRv2 data west of ~95 W and CRU325 trends seem better than CRU401. Trends in PRISM data (1981–2015)^68^ have mostly: increasing q over the Midwest especially Nebraska and the Dakotas, increasing q over the eastern third of the CONUS especially the northeast, and drying over the Rockies, Great Basin, and Southwest. PRISM trends differ markedly from trends shown here over the southwest. However, the vapor pressure remains quite low so the impact on heat stress is small. CRU and PRISM data have better agreement in the northern third of the CONUS. Trends in q over individual decades vary^57^ greatly: 1973–1982 has moistening over the west and drying over the east CONUS that reverses for 1983–1992 and 1993–2002 becoming small for 2003–2015 in HadISDH; q trends over 1975–2010 are positive over all but the southwest.

### Maximum Heat Index Trends

Tmax trends are better captured by CRU401 and moisture trends seem better in CRU325. For the reanalyses, Tmax trends are better in ERA-20C.

**Figure 4:**
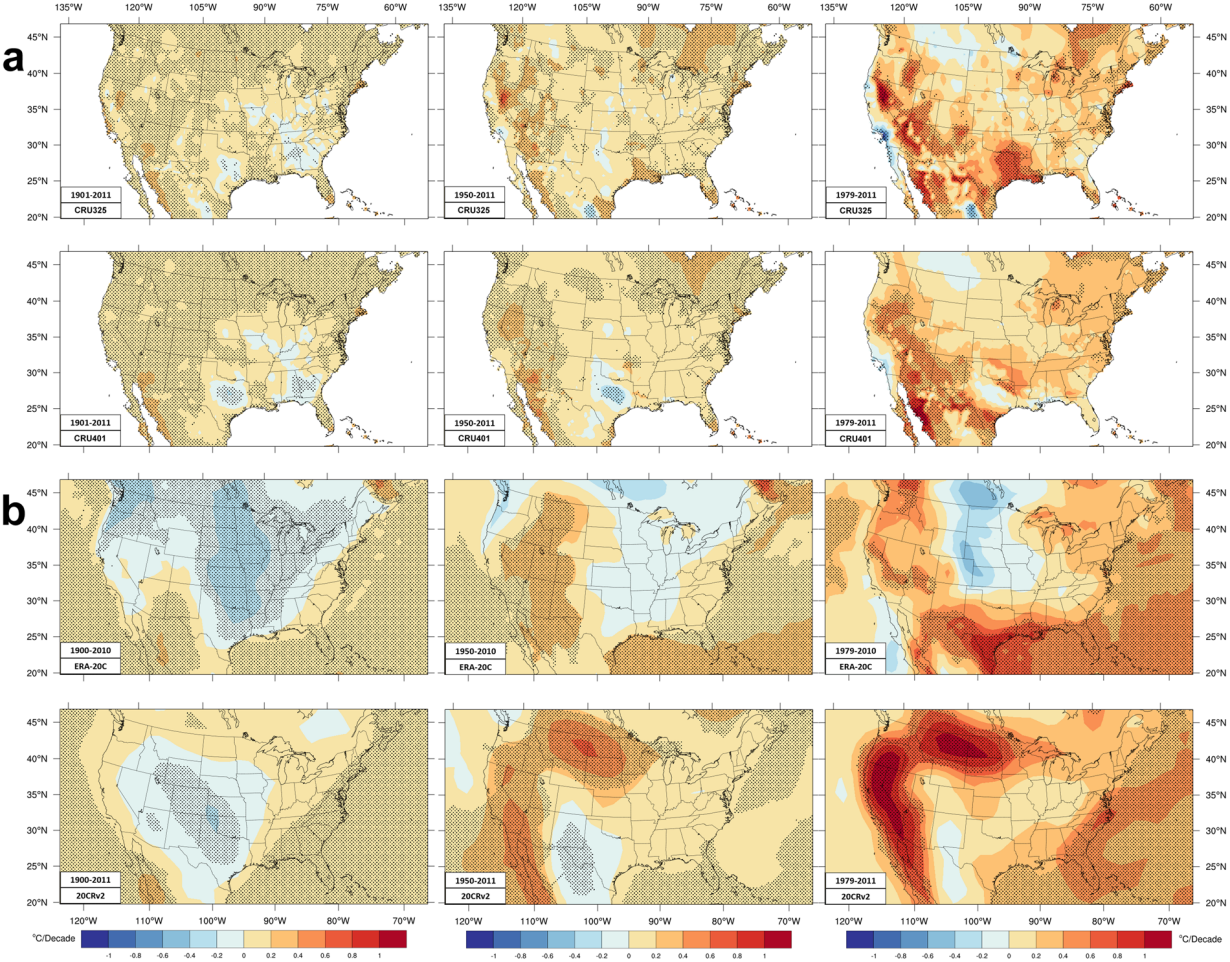
Trends of heat index (HI). Similar format as Fig. 1. JJA average (**a**) daily mean HI and (**b**) daily maximum HI (HImax). The color scale is K/decade.

Figure 4 shows trends of HI; Supplementary Figure S4 shows THI. Figure 4a has daily mean HI trends which should be compared with daily mean temperature (Tm) trends (Supplementary Fig. S3). Figure 4b shows daily maximum HI (HImax).

Trends of Tmax and HI are similar during the longer time period, with notable differences. The Midwest and southern cooling seen in CRU Tmax data is greatly reduced in area for Tm and slightly smaller still for daily mean HI. Significant HI cooling occurs only in small regions of the southeast and Texas. West of ~100 W, Tm and HI have a larger region of significant warming compared with Tmax. The impact of moisture on ERA-20C is less apparent and Tmax trends look very similar to HImax trends. For 20CRv2, the significant cooling area and rate both decreased for HImax compared to Tmax; also, moistening over Florida has reversed the Tmax cooling to be a significant HImax increase.

The intermediate period has no significant region of Tm cooling. Small, but significant cooling regions over northern Texas occur in CRU HI trends due to drying discussed above. The rate and significant area of HI warming west of ~105 W are larger than for Tmax in CRU data. Tm trends do not have the warming hole of Fig. 1. Tmax warming along the eastern seaboard becomes significant warming for Tm and HI. The Tmax warming hole also diminishes for HImax trends in 20CRv2 and ERA-20C. Weak Tmax cooling over the Ohio River valley disappears in 20CRv2 HImax trends. Both reanalyses tend to reduce HI trends relative to Tmax in the northwest. Where 20CRv2 moisture trends are positive in the southwest, the Tmax cooling trends are decreased, but HImax trends are still negative. ERA-20C Tmax warming out west is less for HImax due to decreasing moisture content.

As with Tmax, trends in HI are amplified for the shorter period. Both CRU datasets have much higher HI trends than Tmax because Tm does. Tm and HI have larger areas than Tmax of significant trends in the southwest quadrant and look similar due to the low moisture content. (Cooling near the west coast is ignored; see Tmax discussion.) The central CONUS Tmax warming hole appears in Tm and mean HI only near the Canadian border (NCD did not find cooling there). HI trends elsewhere, especially east of 90 W, are intensified (as is Tm) relative to Tmax. For 20CRv2, drying in western and northern states reduces the prominent Γ-shaped warming in HImax compared to Tmax; but moistening elsewhere makes HImax warming greater than Tmax east of a line from Texas to Wisconsin. In ERA-20C data the cooling hole weakens but does not disappear. The rising moisture over Texas makes HImax warming exceed Tmax warming. Like CRU datasets, increasing moisture over the northeast amplifies HImax trends compared with Tmax trends.

HI trends at 71 southeastern stations (1979–2015)^70^ find: warming over the Carolinas and the Gulf coast near the Mississippi River, but cooling on Florida’s west coast across to the Georgia Atlantic coast; the gridded datasets shown do not match those HI trends well, though CRU325 comes closest. Equivalent temperature from PRISM data (1981–2015)^68^ have: large positive trends over northern and high plains (perhaps most consistent with 20CRv2 trends), higher trends in the northeast (like all four datasets here), positive trends over the northwest (like all four here), and negative trend in the southwest (opposite to the four here) due to strong drying there (contrary to trends here).

## Trends in Reanalyses

Other reanalyses are briefly compared against the main results from CRU and NCD data. These reanalyses incorporate new observing streams as they become available, so they are not intended for long term trends. However, the bias correction and sophisticated data assimilation may make ERA-I useful^27^ for temperature trends. The CONUS has many surface and upper-air stations, but introducing satellite retrievals may impact the climatology of low-level water vapor^71^. Unlike CRU data, near-surface temperature is a reanalysis model product and not all reanalyses assimilate surface temperature observations. NARR does not assimilate surface temperatures as doing so amplified differences from observed values^30^. Direct assimilation of surface temperature and water vapor land station observations by ERA-I^27^ helps explain^72^ higher correlations between ERA-I and CRU values compared with CFSR and a precursor^73^ to MERRA2 during summer.

### Maximum Temperature Trends

**Figure 5:**
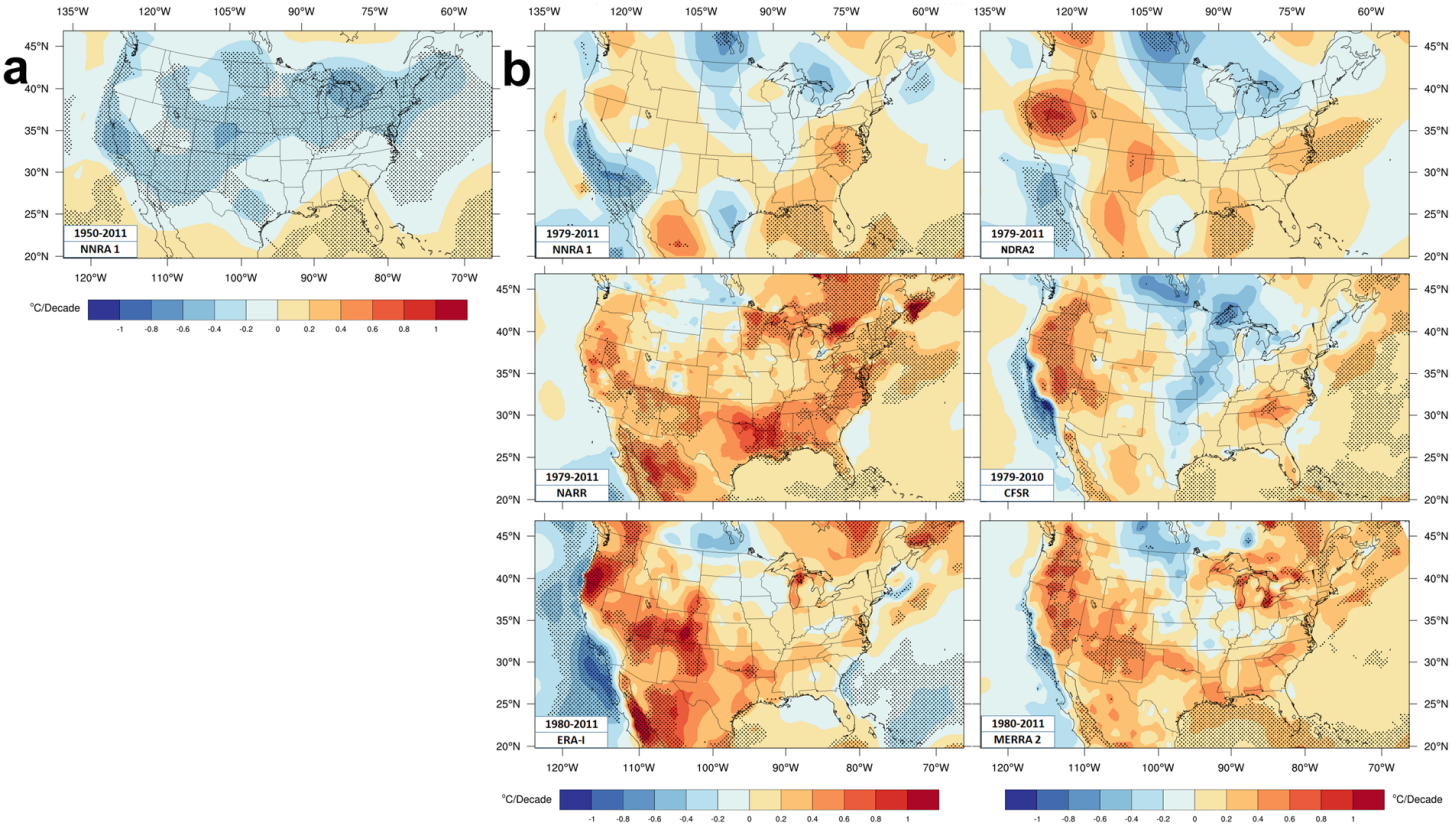
Reanalyses Tmax trends. (**a**) Intermediate time period Tmax trends for NNRA1. (**b**) Shorter time period Tmax trends for six reanalyses.

Figure 5 shows JJA Tmax trends. Compared with CRU data, NNRA1^25^ Tmax trends do not match well CRU or NCD values during the intermediate period. Excessive cooling along the west coast and northern Plains was noted before in 20-year averages^50^.

MERRA2 and ERA-I match Tmax trends in CRU and NCD better than other reanalyses shown; locations and magnitudes of warming regions in the west, across the southern CONUS, and along the east coast; warming magnitudes are greater than for CRU401 and generally closer to NCD values. MERRA2 captures the Midwest warming hole better than ERA-I (including more extensive warming near the Great Lakes) and downplays west coast cooling, better matching NCD. CFSR has the warming hole extending too far north and south. CRU and all reanalyses except 20CRv2 have a warming hole along the central CONUS/Canada border that is not seen in NCD. Tmax extreme statistics (location and scale) trends^74^ for ERA-I and MERRA2 match values over the western CONUS, while MERRA2 has a better pattern of annual mean Tmax trends (1980–2015) over CONUS than ERA-I. Excessive cooling in NNRA1 is a known problem unlike NDRA2 and ERA-I (1979–2010)^64^.

### Moisture Trends

**Figure 6:**
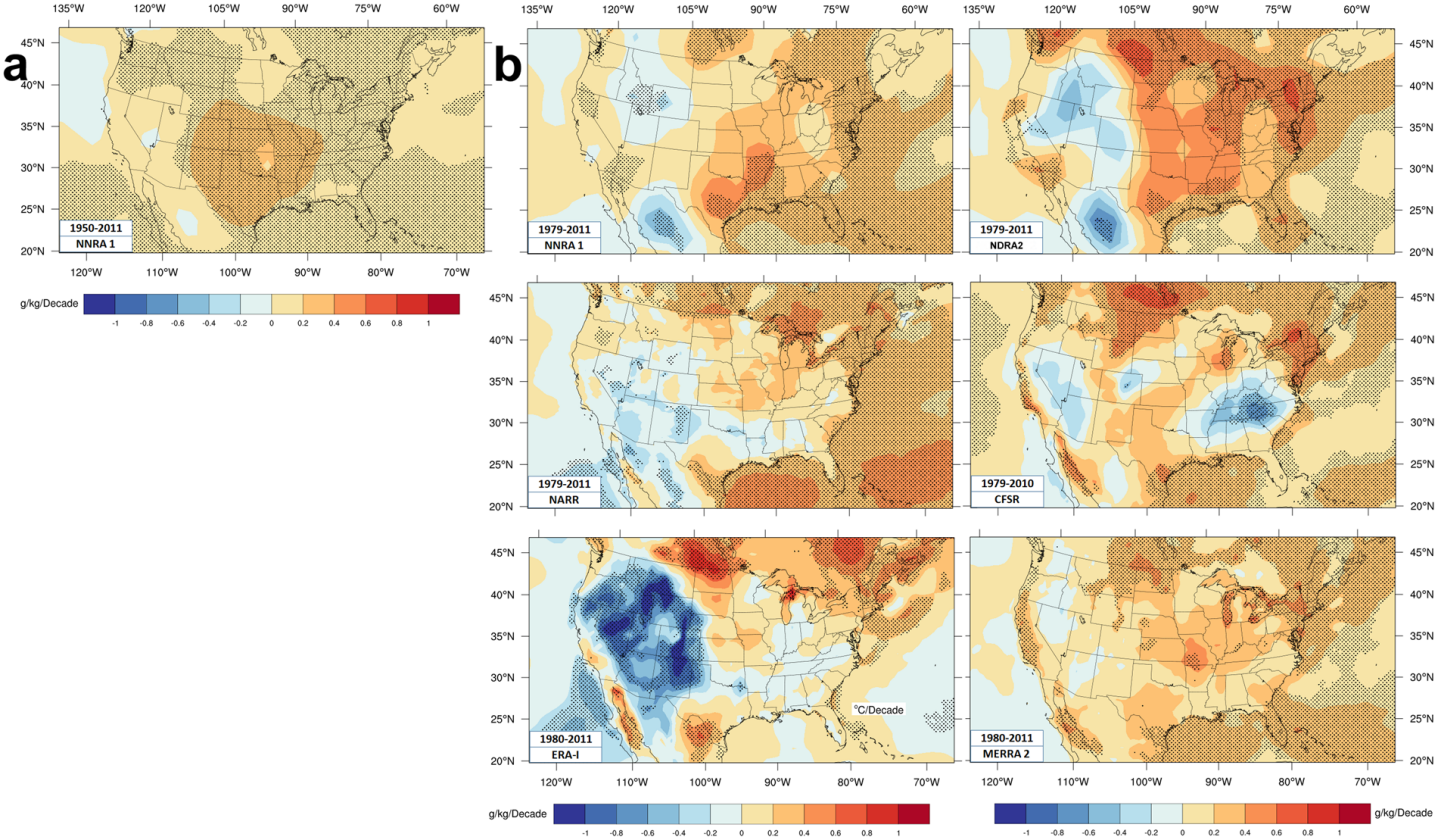
Reanalyses moisture trends. Moisture trends at the time of maximum temperature (**a**) Intermediate time period for NNRA1 and (**b**) shorter time period for six reanalyses. Trends are change per decade of gm water vapor per kg of air except for the bottom center panel. ERA-I trends are of dewpoint at time of Tmax in K/decade. The numbers in the color scales apply to all panels.

Moisture trends at the time of maximum temperature are shown in Fig. 6.

NNRA1 during (1950–2011) moistens most of the CONUS, somewhat like CRU325 trends.

Moisture trends during the shorter time period differ markedly, from each other and from CRU data. These reanalyses favor more drying over the southwest than CRU or 20CRv2, though all reanalyses moisten the northeast and most moisten the Midwest. ERA-I and MERRA2 (1981–2015) q trends^68^ are similar to Fig. 6. Drying over the western half in ERA-I is stronger and more widespread than in other reanalyses in Fig. 6; stronger than the trends (1976–2004)^55^ and (1975–2010)^57^ in surface analyses specifically designed to monitor moisture; but more similar to PRISM trends^68^. Comparing successive decadal averages from 1989–2008 in ERA-I finds^60^ similar results. NNRA1 trends for 1979–2012^75^ are similar to Fig. 6.

### Maximum Heat Index Trends

**Figure 7:**
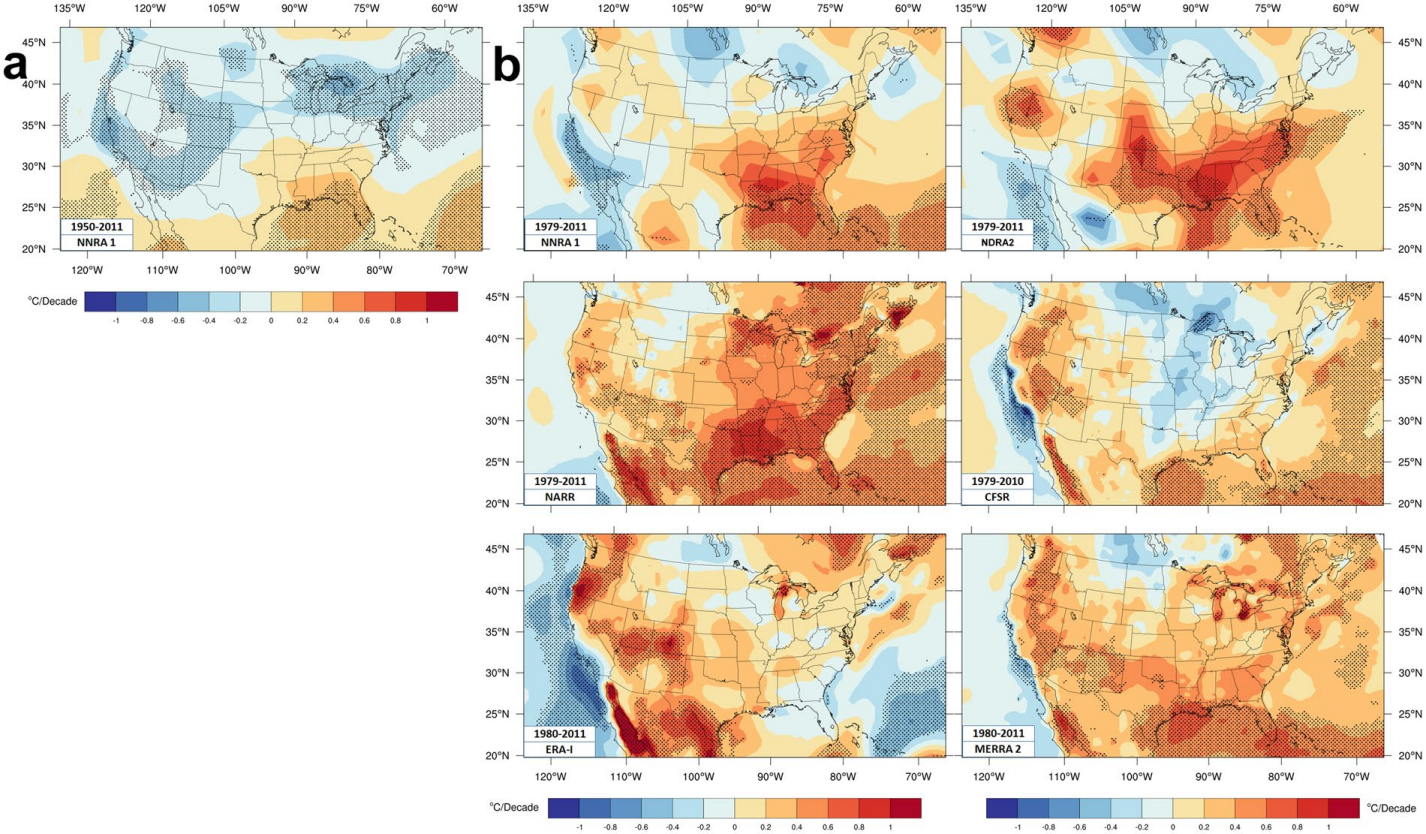
Reanalyses maximum Heat Index Trends. HImax trends (**a**) Intermediate time period trends for NNRA1 and (**b**) shorter time period trends for six reanalyses.

Figure 7 displays HImax trends; Supplementary Fig. S5 has maximum THI trends.

Significant areas of Tmax cooling In NNRA1 (1950–2011) shrink by adding moisture trends so HImax warming expands in the southeast. However, NNRA1 again looks very different from the other trends most places.

Over the shorter period, the Tmax warming hole largely disappears in HImax trends. Moisture had a similar impact on CRU, 20CRv2, and ERA-20C trends. HImax trends exceed Tmax trends over the eastern two-thirds of the CONUS with some exceptions (e.g. southeast in ERA-I, mid-Atlantic states in CFSR). The HImax trend pattern is otherwise similar to the Tmax pattern so ERA-I and MERRA2 trends look more similar to CRU trends.

## Summary Discussion

Moisture elevates stress from high temperatures. Moisture trends are examined by HI changes over three time periods.

Over the longer period, Tmax trends are weak partly due to the warm period of the 1930’s. Even so, Tmax has a warming hole over the Midwest during all time periods in CRU, ERA-20C, and 20CRv2. Warming elsewhere is strongest in the interior west and strong over the Great Lakes and northeast. CRU401 Tmax trends are smoother and have reduced peak values compared to CRU325. CRU401 trends generally match better magnitudes and signs of corresponding NCD trends (especially where passing significance testing) except along west coastal regions. Neither long-term reanalysis matches the NCD pattern well for the longer time period, though ERA-20C may do better east of 105 W. ERA-20C matches NCD better than 20CRv2 during the intermediate and shorter time periods across the Rockies and towards the Gulf coast, while 20CRv2 may match better in the southeast. These datasets (except 20CRv2) place a cooling hole in the Montana and North Dakota region during the shorter time period, but this region has strong warming in NCD.

Guidance from various studies is mixed regarding moisture trends. Opposite trends appear over the southwestern CONUS in datasets examined here and published elsewhere. CRU trends are opposite to PRISM and HadISDH trends in the southwest, but moisture content there is generally low. There is broad agreement among datasets for moistening the middle third of the CONUS that increases towards the north. An exception is over north Texas where CRU401 has drying. Datasets agree in moistening the northeast, and maybe the Gulf Coast, with Florida and Mid-Atlantic States being less certain.

Daily mean and maximum HI trends reduce if not remove entirely the Tmax warming hole. Moisture trends also amplify HI warming over the northeast. Less clear is how moisture changes trends in the southeast as CRU trends disagree about moisture trends, though PRISM and HadISDH q trends are positive there implying greater HI than Tmax warming. Over the southwest, CRU have strong moistening but values remain low so CRU Tm and HI warming are similar and very strong; PRISM and HadISDH have drying, implying slightly less HI warming. Over northern Texas HI cooling occurs in CRU trends.

While tempting to use, some reanalyses are not appropriate for trends even over the observation-rich CONUS. ERA-I and MERRA2 matched CRU and NCD trends better than other short-period reanalyses.

## Methods

### Dataset Properties

Table 1 summarizes key properties of data used for computing and plotting Tmax, q, Td, HI, and THI. Table 1 also has notes about the input variables. There can be indirect effects such as how direct input of precipitation (e.g. NARR, CFSR, MERRA2) can influence near-surface temperature. These and other details are beyond the scope of the intended comparisons and do not easily fit in the table. Similarly, details of the bias correction procedures (e.g. MERRA2 has a near-surface cold bias^28^ that declines over the period) used for reanalyses are beyond the scope of this study. Hence, Table 1 only has basic information, such as ERA-I assimilates station temperatures but NARR does not.

**Table 1:** Properties of the Datasets.

Name	Level	Coverage/time range	Time Res.	Horizontal Res. Grid pts/degrees/distance	Data history	Variables	Special Notes
**Interpolated station data**							
CRU325	Station	Global1901–2011	Monthly	720 × 3600.5°lat × lon	Average of daily values	Tmax, Vap, Tm	Triangulated linear interpolation, Tmax, Tm from observed mean and diurnal range, Vap from mix of observed and ‘synthetic’ values.
CRU401	Station	Global1901–2011	Monthly	720 × 3600.5°lat × lon	Average of daily values	Tmax, Vap, Tm	Angular distance weighting, Tmax, Tm from observed mean and diurnal range, Vap from mix of observed and ‘synthetic’ values.
NCD	Station	CONUS 1900–2011	Monthly	Varies, single value per CD	Average of forecast daily maximum	Tmax	Station data merged within each CD, subject to various geographic and sampling adjustments
**Reanalyses**							
NARR	2 m	Regional1979–2011	3-hourly	349 × 2770.3°32 km	2D field	T, RH	rcm2rgrid used to interpolate to rectangular grid. T from operational forecast system but not 2 m values; 2 m RH from assimilated 3-D fields.
MERRA2	2 m	Global1980–2011	1-hourly	576 × 3610.625° × 0.5°70 × 55 km	Instantaneous 2D Collections, instl1_2d_asm_Nx: Single-Level Diagnostics	T, q, surface pressure	Derived from assimilation of radiosonde and other data, but not 2 m land station observations of T or Vap.
ERA-20C	2 m	Global1900–2010	6-hourly, monthly for ensemble values	320 × 1601.125°125 km	ERA 20th Century atmospheric ‘final’ surface analysis	T, Td	Also has 10 ensemble members. Model-created data from surface: pressure and oceanic winds observations
20CRv2	sig995	Global1900–2011	6-hourly	180 × 912.0°220 km	Regular Gridded Data, monolevel, ensemble mean	T, RH	Also has 56 ensemble members. Model-created data as only surface pressure data observations input.
NNRA 1	sig995	Global1950–2011	6-hourly	144 × 732.5°275 km	3D field	T, RH	Both variables are class ‘B’: direct observation input with ‘strong’ model influence
CFSR	sig995	Global1979–2010	6-hourly	720 × 3610.5°55 km	High Resolution 3D Analysis Pressure Level Data (pgbhnl),	T, RH	NCO used to concatenate files into single file for each year. Does not assimilate surface station values.
NDRA2	1000 hPa	Global1979–2011	6-hourly	144 × 732.5°~275 km	Pressure level data	T, RH	Variables similar to NNRA1
ERA-I	2 m	Global1980–2011	6-hourly	480 × 2410.75°80 km	Instantaneous, temporally varying, surface level analysis	T, Td	Direct assimilation of 2 m station T and Vap plus other data.

Abbreviations: T = air temperature, RH = relative humidity, q = specific humidity, Td = dew point T, Tmax = daily maximum T, Vap = water vapor pressure, CD = NOAA US climate divisions.

### Reanalyses and station data links

CRU325:

- Data download: https://crudata.uea.ac.uk/cru/data/hrg/cru_ts_3.25/
- Data details: http://catalogue.ceda.ac.uk/uuid/c311c7948e8a47b299f8f9c7ae6cb9af

C.RU401:

- Data download: https://crudata.uea.ac.uk/cru/data/hrg/cru_ts_4.01/
- Data details: http://catalogue.ceda.ac.uk/uuid/58a8802721c94c66ae45c3baa4d814d0

NCD:

- Data download: ftp://ftp.ncdc.noaa.gov/pub/data/cirs/climdiv/
- Data details: https://www.ncdc.noaa.gov/monitoring-references/maps/us-climate-divisions.php

20CRv2:

- Data download and details: https://www.esrl.noaa.gov/psd/data/gridded/data.20thC_ReanV2.monolevel.html

CFSR:

- Data download: https://rda.ucar.edu/datasets/ds093.1
- Data details: https://rda.ucar.edu/datasets/ds093.1/docs/CFSR-Hourly-Timeseries.pdf

ERA-20C:

- Data download: https://rda.ucar.edu/datasets/ds626.0/index.html#!access
- Data details: https://rda.ucar.edu/datasets/ds626.0/

ERA-I:

- Data download: http://apps.ecmwf.int/datasets/data/interim-full-daily/levtype = sfc/
- Data details: https://www.ecmwf.int/sites/default/files/elibrary/2011/8174-era-interim-archive-version-20.pdf

MERRA2:

- Data download: https://gmao.gsfc.nasa.gov/reanalysis/MERRA-2/data_access/
- Data details: https://gmao.gsfc.nasa.gov/pubs/docs/Bosilovich785.pdf

NARR:

- Data download: ftp://ftp.cdc.noaa.gov/Datasets/NARR/monolevel
- Data details: https://www.esrl.noaa.gov/psd/data/gridded/data.narr.monolevel.html

NDRA2:

- Data download/details: https://www.esrl.noaa.gov/psd/data/gridded/data.ncep.reanalysis2.pressure.html

NNRA 1:

- Data download/details: https://www.esrl.noaa.gov/psd/data/gridded/data.ncep.reanalysis.surface.html

### HI and THI methodology

HI and THI are found as follows.

1. Download near-surface temperature and moisture data.
2. Compute HI and THI using the temperature and moisture variables for all points in time and all grid points using the appropriate equations. Then write out HI and THI to separate netCDF files.
  - For CRU data, vapor pressure (Vap) is first converted to relative humidity (RH) by defining the saturation vapor pressure (VapS) from daily mean temperature T_M_ (in K):
    $$VapS=6.11\times {10}^{(\frac{7.5\times ({T}_{M}-273.15)}{237.3+({T}_{M}-273.15)})}$$
    RH = (Vap/VapS) × 100. Dewpoint *Td* in °C is calculated using:
    $${T}_{d}-273.15=Td=\frac{237.3(\frac{\mathrm{ln}(\frac{RH}{100})}{17.27}+\frac{({T}_{M}-273.15)}{237.3+({T}_{M}-273.15)})}{1-(\frac{\mathrm{ln}(\frac{RH}{100})}{17.27}+\frac{({T}_{M}-273.15)}{237.3+({T}_{M}-273.15)})}$$
    where: T_d_ and T_M_ are in K.
  - For CRU data and reanalyses with T and RH:
    $$THI={T}_{F}-(0.55-(0.55\times RH\times 0.01)\times ({T}_{F}-58))$$
    The HI equation
    $$\begin{array}{rcl}HI & = & -42.379+2.04901523\times {T}_{F}+10.14333127\times RH-0.22475541\times {T}_{F}\times RH\\  &  & -6.83783x{10}^{-3}\times {{T}_{F}}^{2}-5.481717\times {10}^{-2}\times R{H}^{2}+1.22874\times {10}^{-3}\times {{T}_{F}}^{2}\times RH\\  &  & +\,8.5282\times {10}^{-4}\times {T}_{F}\times R{H}^{2}-1.99\times {10}^{-6}\times {{T}_{F}}^{2}\times R{H}^{2}\end{array}$$
    is discussed here: http://www.wpc.ncep.noaa.gov/html/heatindex_equation.shtml and is valid from 20–50 C. T_F_ is temperature in degrees Fahrenheit, same units for HI.
  - For reanalyses with T, specific humidity, and pressure:
    First compute RH using:
    $$RH=0.263\times P\times q\times {({e}^{\frac{17.67\times ({T}_{K}-273.16)}{{T}_{K}-29.65}})}^{-1}$$
    where: T_K_ is in K. Then use the formulas above to compute HI and THI.
  - For reanalyses with T and T_d_:
  $$THI=(0.55\times {T}_{F})+(0.2\times {T}_{d})+17.5$$
  T and T_d_ were used to compute RH then to compute HI using the formula in b.
  $$RH=100\times \frac{{e}^{\frac{17.625\times ({T}_{d}-273.15)}{243.04+({T}_{d}-273.15)}}}{{e}^{\frac{17.625\times ({T}_{K}-273.15)}{243.04+({T}_{K}-273.15)}}}$$
  $$\begin{array}{c}RH=relative\,\,humidity\,in\, \% \\ P=pressure\,in\,Pascal\\ q=specific\,humidity\,in\,kg/kg\end{array}$$
3. Compute the daily extrema using polynomial fitting (see below) and write out to separate netCDF files.
4. Use NCO to extract June-August data for Tmax, HI (mean or maximum) and THI (mean or maximum), compute the JJA average for each year, and write out to separate netCDF files.
5. Compute linear trends for various time periods using linear least-squares fitting (regCoef is the NCL function). Then plot the trends.

### Ensemble spread methodology

20CRv2 data include the ensemble spread as a variable. For ERA-20C, monthly mean temperature values were converted to seasonal means for each year in each ensemble member. The standard deviation was calculated for each year. These standard deviations were averaged to obtain the top row of Fig. 2a and their trend forms the top row of Fig. 2b.

### Maximum value methodology

Daily maximum temperature must be calculated from reanalysis data. The procedure for estimating the absolute extrema (maxima and minima) of a quartic (4^th^ power) polynomial is as follows:

1. Compute the derivative.
  $$f(x)=e{x}^{4}+f{x}^{3}+g{x}^{2}+hx+i$$
  $$f^{\prime} (x)=4e{x}^{3}+3f{x}^{2}+2gx+h$$
  Coefficients: *a* = 4*e*, *b* = 3*f*, *c* = 2*g*, *d* = *h*
2. Set the derivative equal to zero.
  $$f^{\prime} (x)=0$$
3. Solve for the critical points.

- The general solution to the cubic equation:
  $$a{x}^{3}+b{x}^{2}+cx+d=0$$
  $${x}_{k}=-\frac{1}{3a}(b+{\zeta }^{k}C+\frac{{{\rm{\Delta }}}_{0}}{{\zeta }^{k}C}),k\in \{0,1,2\}$$
  $${{\rm{\Delta }}}_{0}={b}^{2}-3ac$$
  $${{\rm{\Delta }}}_{1}=2{b}^{3}-9abc+27{a}^{2}d$$
  $$C=\,\sqrt[3]{\frac{{{\rm{\Delta }}}_{1}\pm \sqrt{{{\rm{\Delta }}}_{1}^{2}-4{{\rm{\Delta }}}_{0}^{3}}}{2}}$$
  Choosing the sign:
  If Δ_0_ ≠ 0, either sign may be chosen.
  If Δ_0_ = 0, choose the sign such that the two terms inside the cube root do not cancel.

$$\zeta =-\frac{1}{2}+\frac{1}{2}\sqrt{3}i$$

Real root solutions:

First calculate the discriminant: Δ = 18*abcd* − 4*b*^3^*d* + *b*^2^*c*^2^ − 4*ac*^3^ − 27*a*^2^*d*^2^

- If Δ > 0, then the equation has three distinct real roots.

- Procedure: Use the general solution above. The imaginary parts in C and *ζ* cancel each other.

- If Δ = 0, then the equation has a multiple root and all its roots are real.

- Procedure:

- One root scenario (triple root): Δ = 0 and Δ_0_ = 0

Example: (*x*−1)^3^ = 0

$$x=-\frac{b}{3a}$$

Two root scenario (double root): Δ = 0 and Δ_0_ ≠ 0

Example: (*x* − 1)^2^ (*x* + 2) = 0

$${x}_{1}=\frac{9ad-bc}{2{{\rm{\Delta }}}_{0}}$$

$${x}_{2}=\frac{4abc-9{a}^{2}d-{b}^{3}}{a{{\rm{\Delta }}}_{0}}$$

- If Δ < 0, then the equation has one real root and two non-real complex conjugate roots.

- Procedure: Use the solution below:

$$x=-\frac{1}{3a}(b+C+\frac{{{\rm{\Delta }}}_{0}}{C})$$

This is the same as the general solution but with *ζ*^*k*^ removed since *k* = 0.

4. Substitute the critical points and end points into the quartic polynomial.
5. The largest value is the maximum and the smallest value is the minimum.

Note: The procedure above was used for all reanalyses, regardless of their temporal resolution. For reanalyses with a temporal resolution of higher than 4 times per day, a quartic polynomial fit was used to fit all of the points in time for each day. The hourly MERRA2 data have 25 points per day (including endpoints). The quartic fit was applied separately to hours 0, 1, 2, 3, 4; to hours 1, 2, 3, 4, 5; to hours 2, 3, 4, 5, 6; and so on – 21 times per day. The extrema for that day are the largest value and the smallest value from all 21 fits. A quartic fit for more than 5 points for each day will result in an imperfect fit; a 24^th^ power polynomial would have been needed for MERRA2 data for a perfect fit.

MERRA2 includes Tmax as a variable and trends of that variable match Tmax trends based on our quartic procedure. (Since we do not know the time of Tmax provided by MERRA2, we need our quartic procedure to obtain HImax.) MERRA2 data have the finest time resolution so the procedure for estimating the maximum temperature is likely more accurate than having data just four times daily. Also, the available data are at different near-surface levels making direct comparison of values inappropriate but comparison of trends may still be reasonable. A second test examined how trends differ when using Tmax values estimated from the four times daily data versus estimated Tmax values at a different near-surface level. NDRA2 also includes forecast maximum temperatures at 2 m. Trend maps using our scheme (four times daily T values at 1000 hPa; Fig. 5) and the forecast 2 m maximum are similar in magnitude and spatial patterns with few exceptions. The strong similarity between these disparate quantities validates making qualitative reanalyses trend comparisons here.

### Statistics

It is generally not sufficient to employ a least squares estimation of the trend for a time series^76^. The error associated with such estimation can be significant. A number of assumptions must be met in order for the trend estimation to be valid. The noise for each data point must be assumed independent and identically distributed as normal but this cannot be the case if cyclical or seasonal effects exist or if the residuals are autocorrelated. The time series should be stationary, a condition in which the probability distribution of the time series is constant over time.

Trends were calculated for the JJA mean values of daily mean and daily maximum T, THI, and HI for the three time periods. These trends were computed using least squares estimation (LSE). Taking seasonal means causes autocorrelation between residuals to be greatly diminished and therefore greatly simplifies the required computations. Because the annual cycle was no longer present in the JJA mean values, the seasonal cycle could be neglected. Cyclical, inter-annual or multi-decadal effects such as the North Atlantic Oscillation and the Pacific Decadal Oscillation were weak or non-existent over long time periods, and therefore the seasonality component could be entirely neglected^16^. Since each value of the JJA data points was separated by near a year without seasonality, it was reasonable to assume uncorrelated residuals under the least squares estimation. Since the desire is to understand whether the climate is gradually warming or cooling over time, the use of a linear slope is appropriate. Therefore, LSE is appropriate for determining trends of seasonal means and its use is consistent with studies that involve averages over long time periods^12,77^.

The Mann Kendall test was used to determine whether the T, Td, HI, THI, and q consistently increased or decreased over time. More specifically, Mann Kendall was used to test whether the linear trends were significantly different from zero^78,79^. The test requires that the data points should not be correlated with each other and that the time series should not exhibit periodic fluctuations. For this study, the test was conducted for trends obtained from LSE. Under LSE, the test was simply applied to the time series. The statistical tests and other steps to prepare the data and to calculate the linear trend used tools in NCL.

## Electronic supplementary material

Supplementary Information
