# Supplementary material for: Contiguous US summer maximum temperature and heat stress trends in CRU and NOAA Climate Division data plus comparisons to reanalyses
Source: Sci Rep. 2018 Jul 24;8:11146. doi: 10.1038/s41598-018-29286-w (PMC6057947; doi:10.1038/s41598-018-29286-w)
Supplement: Supplementary file 1 — Supplementary Information [file 41598_2018_29286_MOESM1_ESM.pdf]

Supplementary Materials for:

Contiguous US summer maximum temperature and heat stress trends in CRU and NOAA Climate Division data plus comparisons to reanalyses

By Richard Grotjahn and Jonathan Huynh

GHCN Monthly Mean Maximum Temperature Trends

Trends of monthly mean Global Historical Climatological Network (GHCN) Tmax station data are shown in Fig. S1. Trends are small during the longer period, generally <0.2 C/decade in magnitude, over most of the CONUS. Just 22 stations have warming between 0.2-0.4 C/decade, scattered mainly in the west.

The station data for the intermediate period continue trends (cooling and warming) seen in the longer time period over central and eastern CONUS, respectively. Warming around the Great Lakes and southeast has developed. With nearly twice as many stations reporting, the intermediate period samples the western half much better now and reveals warming over nearly all of the western third of the US, with amounts >0.4 C/decade over the western interior.

During the shorter time period the station trends are often larger and more spatially-consistent. Warming covers most of the US with the major exception being the northern plains where cooling occurs about a diagonal line from the Montana-North Dakota border to southeastern Missouri. The southwestern CONUS interior has consistent warming with many stations warming >0.8 C/decade. While some individual stations have cooling, most stations in the southeast and Atlantic States have warming.

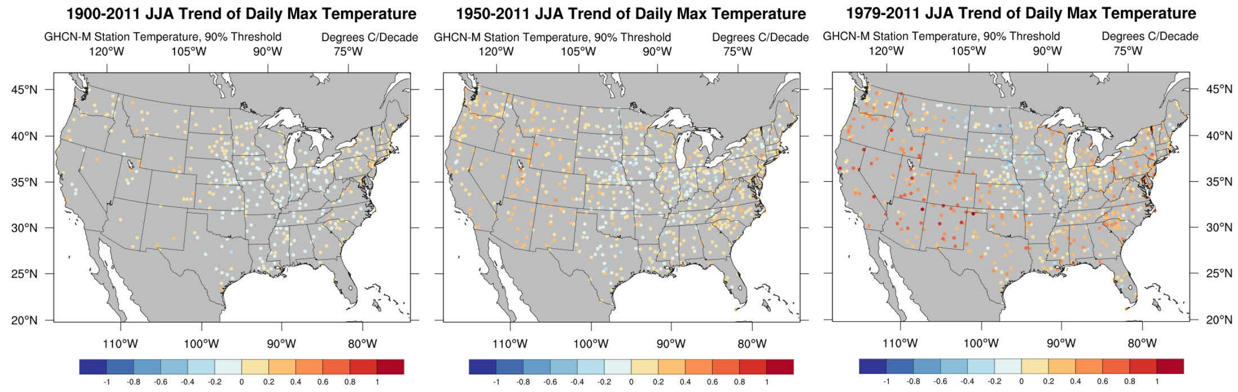

Figure S1. GHCN trends. Summertime trends in C/decade calculated from monthly mean Tmax data at GHCN stations that report at least 90% of the time period. Longer (left panel), intermediate (middle panel), and shorter (right panel) time periods are as in the main text.

Daily Mean CRU Summer Vapor Pressure

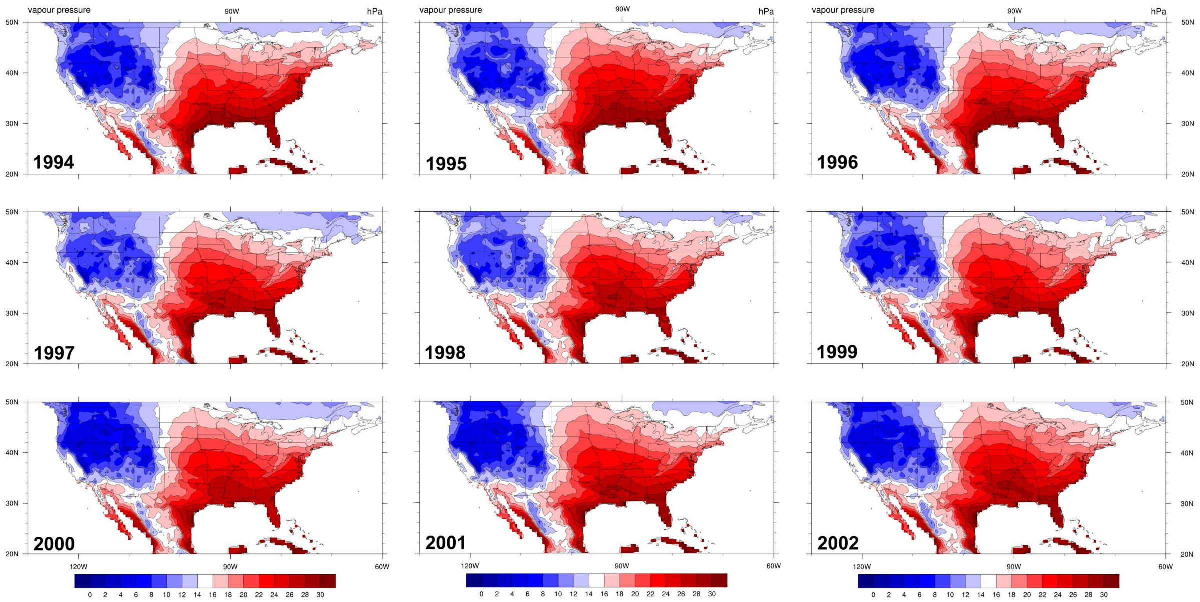

Figure S2. Seasonal averages of daily mean vapor pressure. Summertime averages of vapor pressure, in hPa in CRU401 data.

Daily Mean CRU Temperature Trends

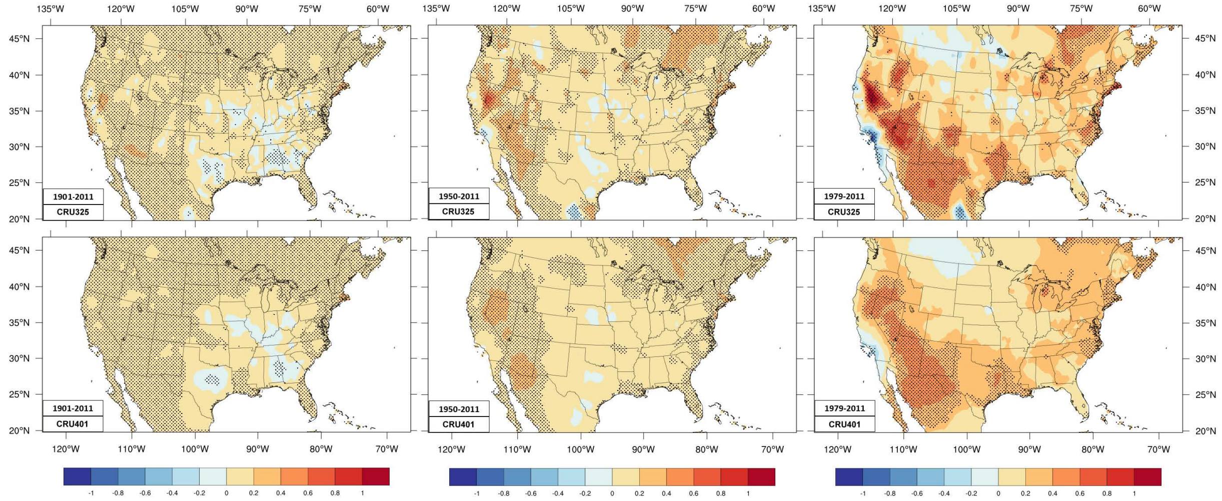

Figure S3. Daily mean temperature trends. Summertime trends in C/decade of seasonal average daily mean temperature, Tm in CRU data.

THI Trends

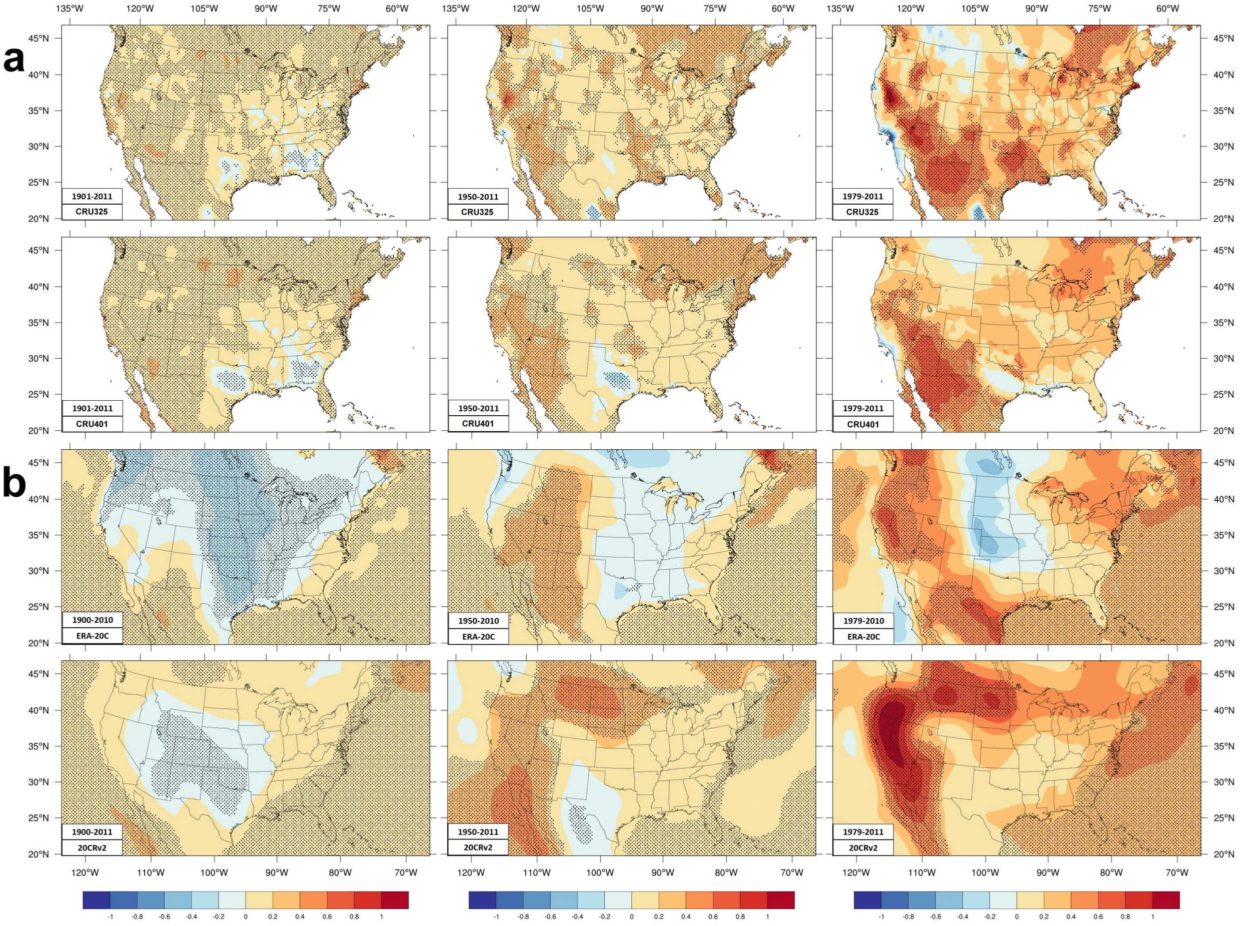

Figure S4. Similar to Fig. 4. THI trends. Seasonal mean JJA trends in C/decade of (a) daily mean THI in CRU data and (b) daily maximum THI in ERA-20C and 20CRv2 data.

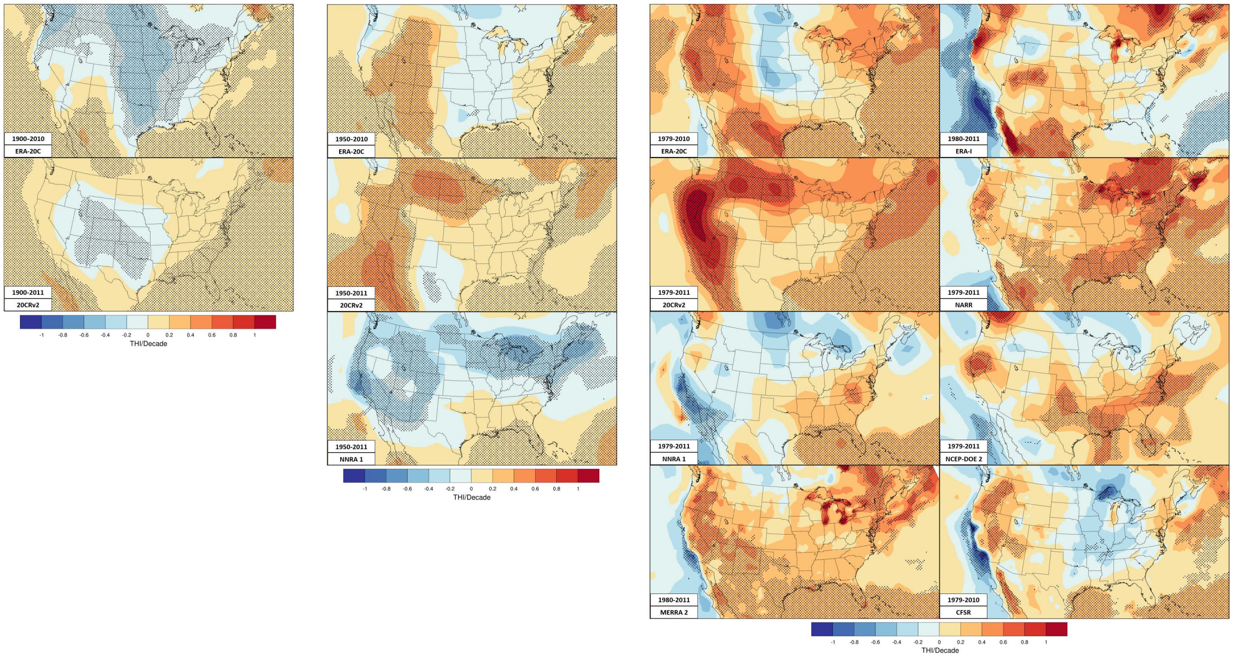

Figure S5. Similar to Fig.7. THI trends in reanalyses. Seasonal mean JJA trends in C/decade of daily maximum THI in the indicated reanalyses.
